# Remimazolam for Sedation and Trachospray for Topicalization During Flexible Nasal Intubation in a Spontaneously Breathing Patient

**DOI:** 10.7759/cureus.77406

**Published:** 2025-01-13

**Authors:** Loes Bruijstens, Rebecca Koch, Raymond Van Der Wal, Lucas Van Eijk, Jörgen Bruhn

**Affiliations:** 1 Anesthesiology and Pain and Palliative Medicine, Radboud University Medical Center, Nijmegen, NLD

**Keywords:** airway topicalization, awake tracheal intubation, flexible bronchoscopy, remimazolam, trachospray

## Abstract

When a difficult airway is anticipated, awake tracheal intubation can be considered. Usually, low doses of sedatives are administered during this procedure for minimal sedation and anxiolysis, such as midazolam and remifentanil. The newly developed ultra-short-acting benzodiazepine remimazolam has a pharmacokinetic profile that is more suitable for titration during awake tracheal intubation than the long-acting midazolam. It can be titrated to effect while spontaneous breathing is preserved. When desired, cessation of administration will result in emergence from sedation in several minutes, but it can also be antagonized with flumazenil if needed.

Here we report a case of a difficult airway in which awake nasal flexible intubation was performed using remimazolam for sedation: background infusion of 0.25 mg/kg/h with an initial bolus of 0.1 mg/kg and later extra boli of 0.04 mg/kg if needed. This was supplemented with a low dose of remifentanil (2 mcg/kg/hr). In addition, topical anesthesia of the upper airway was performed using a specifically designed device called the Trachospray, which nebulizes lidocaine into a fine mist that precipitates in the upper airway of the patient upon inhalation. About 4 ml of lidocaine 4% were inhaled via the Trachospray. An additional 2 ml of lidocaine 2% was sprayed via an epidural catheter advanced through the working channel of the endoscope for further topicalization of the epiglottis, vocal cords, and proximal portion of the trachea. This combination led to adequate anxiolysis and sedation, preservation of spontaneous breathing throughout the procedure, good conditions for successful intubation, and amnesia for the endoscopy and intubation. Further research is needed to establish the superiority of any of these techniques in comparison to other accepted methods.

## Introduction

During awake tracheal intubation, low doses of sedatives are commonly used to comfort patients. Balancing adequate sedation and anxiolysis while avoiding apnea and hypercapnia remains challenging. The Difficult Airway Society (DAS) guidelines for awake tracheal intubation recommend minimal sedation with midazolam and remifentanil [1]. However, midazolam is not an ideal sedative because of its long half-life. Recently, the ultra-short-acting benzodiazepine remimazolam has been introduced for the induction of general anesthesia and procedural sedation [2]. Remimazolam’s sedative effect peaks approximately 3-3.5 minutes after administration, and it is efficiently metabolized via hepatic carboxylesterase-1 to the inactive CNS7054 metabolite, which is then glucuronidated, hydroxylated, and excreted [3]. Similar to midazolam, remimazolam can also be reversed with flumazenil. Its pharmacokinetic profile renders it highly suitable for sedation during awake tracheal intubation.

In addition, for tracheal intubation of a spontaneously breathing patient, excellent topical anesthesia of the airway is key. Although there is no gold standard technique to anesthetize the airway, several techniques are accepted for topicalization [1]. Recently, a new device was developed with the specific goal of upper airway topicalization, called Trachospray (Medspray Anesthesia B.V., Enschede, The Netherlands) [4]. It is a device that is held between the lips by the patient and connected to a 2-cc syringe with a local anesthetic. It creates a soft mist spray of local anesthetic, with a specific size of droplets that are deposited in the mouth, hypopharynx, and vocal cords during spontaneous breathing, with only a small fraction being deposited in the lung, thereby facilitating intubation in a quick and easy manner [5].

In this case report we describe the combined use of remimazolam for anxiolysis and sedation and Trachospray for airway topicalization to enable nasal flexible intubation in a spontaneously breathing patient with a known difficult airway. Review of these cases and presentation in this format followed the guidelines of the Institutional Review Board of Radboud University Medical Center (Nijmegen, the Netherlands).

## Case presentation

A middle-aged female was scheduled for laser resection of a residual tumorous lesion of the palatum and on the floor of the mouth in December 2022. Her medical history revealed a hemi-maxillectomy for a squamous cell carcinoma of the mouth a few months earlier. The tongue and oral cavity had been reconstructed using a free radial forearm flap. The operation had been uneventful, and general anesthesia was uncomplicated, with a grade I Cormack and Lehane intubation classification. However, some days after the operation, the patient presented with sudden intraoral arterial bleeding, for which she underwent nasal flexible tracheal intubation under light propofol sedation and topicalization by "spray as you go" to enable emergency surgery. This intubation was successful, yet the patient had experienced the intubation procedure as traumatic, as she had full remembrance of the intubation. For this newly scheduled surgical procedure, she was again screened and counseled by an anesthesiologist.

The preoperative airway assessment now showed a limited mouth opening of approximately 2.5 cm, a Mallampati score III, an upper lip bite test III, normal thyromental distance, normal neck circumference and extension, and a palpable thyroid cartilage, cricoid cartilage, and cricothyroid membrane. She had a partial dental prosthesis on the upper right side and no teeth or prosthesis in the mandible. Intraorally, the previously inserted radialis flap reconstruction was visible and partially obstructing the view as it was thicker than a normal tongue would be.

Based on the Canadian Airway Focus Group (CAFG) guidelines [6], this patient had risk factors for difficult mask ventilation, ventilation via supraglottic airway, intubation after direct or video laryngoscopy, and extubation. Therefore, flexible nasal intubation under topical anesthesia and sedation was agreed upon with the patient. To minimize anxiety and stress, ensure spontaneous breathing during the procedure, and allow for a quick recovery if the procedure needed to be aborted, the newly available ultra-short-acting benzodiazepine remimazolam was chosen as the primary anxiolytic. Special attention was also given to optimal topicalization of the airway.

**Figure 1:**
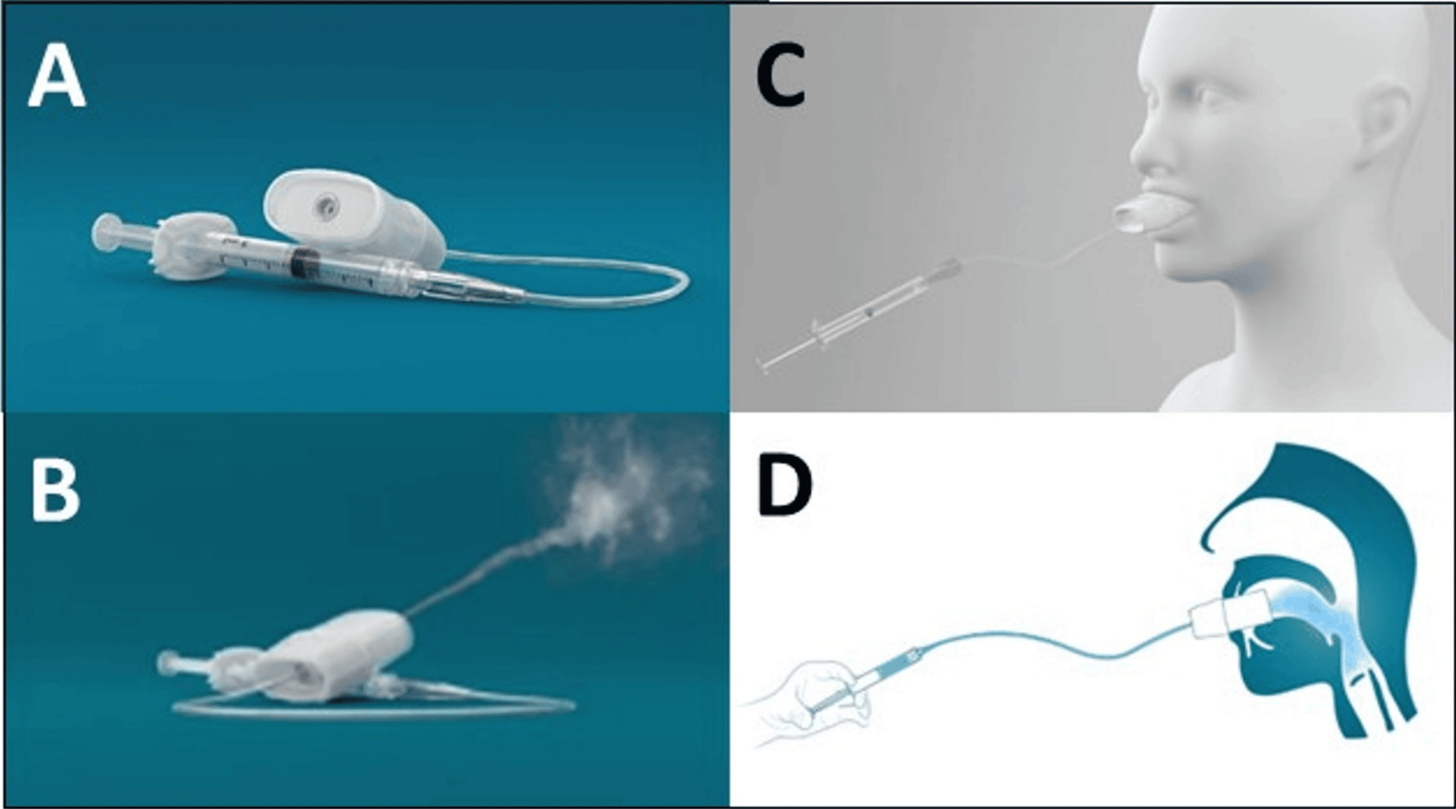
The Trachospray device Local anesthetics are nebulized to anesthetize the upper airway. Credit: Permission to reproduce the images in panels A-D has been obtained from MedSpray Anesthesia B.V.

At the start of the procedure, the patient was optimally positioned in a supine position, head up, and connected to the monitor to assess vital signs. Supplemental oxygen was administered through a nasal cannula, and end-tidal CO_2_ was monitored. Sedation with remimazolam was initiated as a continuous infusion at a rate of 0.2 mg/kg/hr. Xylometazoline (0.5 ml) was nasally administered in the right nostril using an atomizer device, as this was the preferred side for intubation. For topical anesthesia of the upper airway, a Trachospray device (Medspray Anesthesia B.V., Enschede, The Netherlands) (Figure 1) was utilized [5].

**Figure 2:**
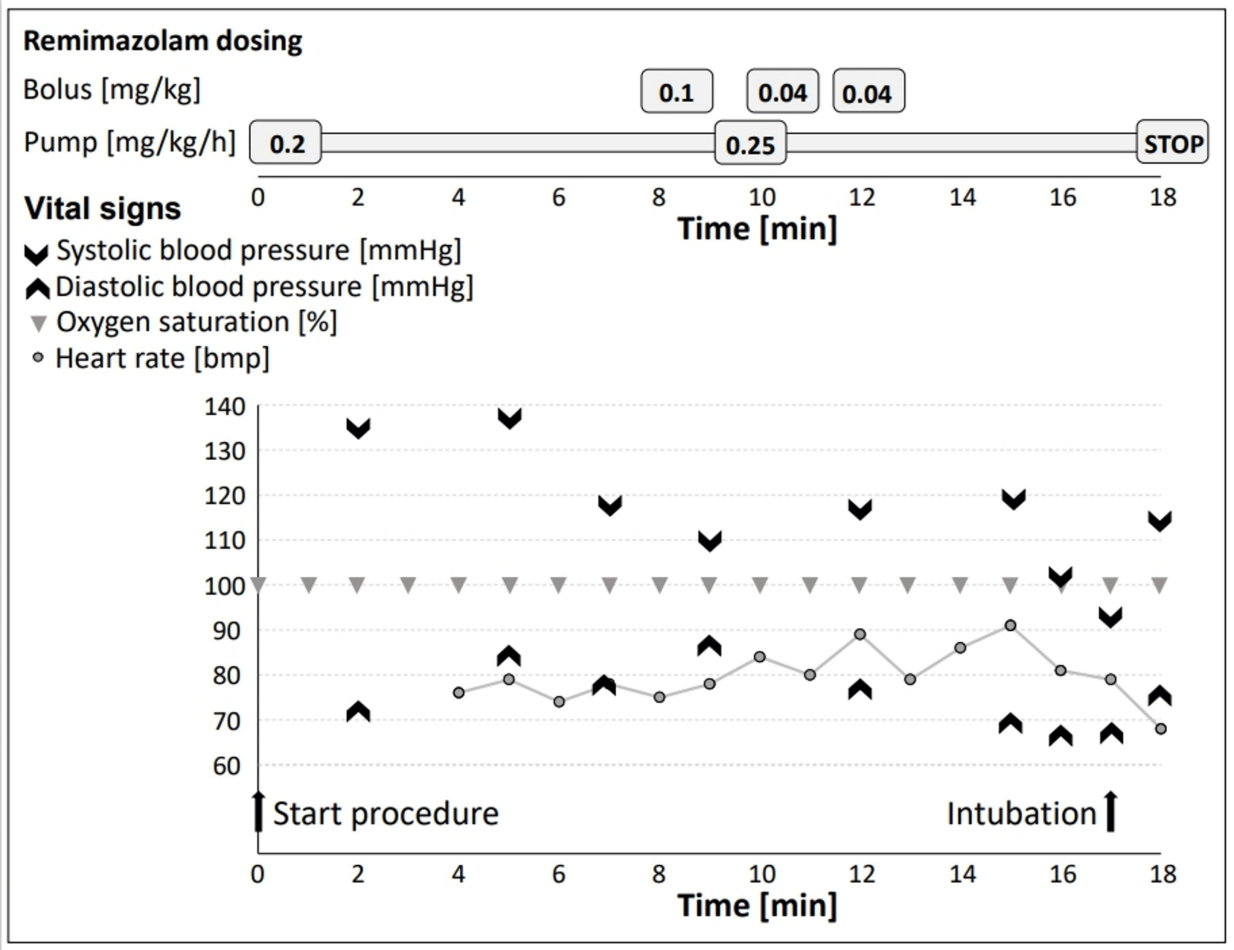
Remimazolam dosing and vital signs over time Dosing of remimazolam overtime during the intubation procedure. Administration was performed both via continuous infusion and additional boli to titrate sedation. Vital signs shows no desaturation and normal values for blood pressure and heart rate.

Four ml of lidocaine 4% was administered over several minutes through the Trachospray. Additionally, 0.5 ml of lidocaine 4% was administered to the right nostril via a nasal atomizer. Then a bolus of remimazolam 0.1 mg/kg was administered, the continuous dose was increased to 0.25 mg/kg/hr, and a low dose of remifentanil (2 mcg/kg/hr) was started to aid in dampening the intubation stimulus. A nasopharyngeal airway device lubricated with 2% lidocaine gel was easily inserted to confirm the patency of the right nostril and assess the patient’s response, which showed no discomfort. Subsequently, a flexible bronchoscope was introduced via the right nostril, and visualization of the glottis was quickly achieved. Another 2 ml of lidocaine 2% was administered through an epidural catheter that was advanced through the working channel of the flexible scope for further topicalization of the epiglottis, vocal cords, and proximal portion of the trachea by "spray as you go." Sedation depth was reassessed, and sequential remimazolam boluses of 2 mg (0.04 mg/kg) were administered at 2-3 minute intervals until adequate sedation was reached. The targeted adequate depth of sedation was “-4 Deep sedation” according to the Richmond Agitation Sedation Scale (RASS): No response to voice, but movement or eye opening to physical stimulation. It was ensured that spontaneous breathing was preserved. Then a nasal tube size 6 could easily be advanced through the nose into the trachea. The procedure did not cause stress, such as visible discomfort or significant changes in blood pressure or heart rate, nor did it lead to signs of excessive sedation, hypotension, or apnea (Figure 2). Correct positioning of the tube was verified using the flexible scope, and capnography reconfirmed proper ventilation in the still spontaneously breathing patient. Sedation was then deepened to general anesthesia using propofol and remifentanil while remimazolam was stopped. From the start of the remimazolam administration at the beginning of the procedure to the end of intubation, 17 minutes had passed (Figure 2).

Following an uneventful surgery, the patient was extubated successfully while awake and in an upright position. When asked about the procedure, the patient recalled the topicalization of the airway and nose but did not perceive it as stressful, and she had no recollection of the remainder of the intubating procedure.

A couple of weeks later, the same patient was presented for a similar procedure. The patient was asked about her experience from the last time and was still pleased with how it had gone and was motivated to have the intubation done in the same manner. This time, the procedure was done in the same sequence, only no remifentanil was used, and only 1 ml of 1% lidocaine was sprayed via epidural catheter. Again, there was an uneventful endoscopy and intubation. As no large differences were noted between the two intubating procedures, one may suggest that the addition of remifentanil is not necessary.

## Discussion

Here we report the use of the ultra-short-acting benzodiazepine remimazolam for sedation and anxiolysis in a patient with an anticipated difficulty on more than two aspects of airway management. In addition, we used the Trachospray device, which is designed with the specific aim of optimally anesthetizing the upper airway for intubation. This combination resulted in adequate anxiolysis and amnesia during intubation while maintaining spontaneous breathing throughout the procedure without relevant hemodynamical changes. There were good circumstances for both the patient and the team.

A potentially difficult airway should be adequately managed to reduce the associated risks. Although a “cannot intubate, cannot oxygenate” scenario is relatively rare, more than half of the severe injuries after anesthesia are still caused by failed intubation or a misplaced endotracheal tube [7]. Therefore, several societies, such as the DAS and the CAFG, have published guidelines to manage airways. The CAFG guidelines from 2021 published a flowchart that helps in decision-making when to consider intubation in a spontaneously breathing patient [6]. Basically, these guidelines advocate the use of local anesthetization for the upper airway mucosa and the administration of sedatives while preserving spontaneous breathing. With regard to sedation, the DAS guidelines advise the cautious use of a combination of remifentanil and midazolam, as both are reversible with specific medication [1]. We used remimazolam instead of midazolam, as remimazolam is much shorter acting, which facilitates (up)titration. To keep a relatively constant level of remimazolam, we also started a low-dose continuous remimazolam infusion after the initial remimazolam bolus. Although remimazolam is usually administered per bolus, previous studies have shown that it is highly suitable for continuous administration [8-10]. Like midazolam, remimazolam can also be reversed with flumazenil. Therefore, we considered it to be safe to strive for a deeper level of sedation in this patient, resulting in even greater comfort for the patient and a higher probability of the desired amnesia, while hypertension due to anxiety, as well as hypotension due to oversedation, are being avoided. Recently two other case reports were published about the use of remimazolam for the management of a difficult airway with a flexible bronchoscope [11,12]. Interestingly, in both case reports, repetitive boluses of remimazolam were used without continuous administration of remimazolam. In our opinion, the continuous administration of remimazolam with a more constant plasma concentration is advantageous in this setting. As with the dosing of 0.25 mg/kg/h remimazolam in the here-reported case, two more additional boluses of remimazolam were needed. We increased the continuous dosing of remimazolam in subsequent cases to 0.5-0.75 mg/kg/h with even better sedation without airway obstructions or apneas. Nonetheless, in each individual case, the targeted depth of sedation should be carefully chosen depending on airway compromise and desired patient cooperation.

This study is limited by the fact that no direct comparisons with other anesthetics can be made. We chose remimazolam based on its beneficial pharmacokinetic profile, while traditionally (combinations of) midazolam, remifentanil, propofol, or dexmedetomidine are mostly used. In this case, the patient had an earlier experience with propofol sedation during awake tracheal intubation in an emergency setting, which she had experienced as traumatic. In our experience, remimazolam is superior to propofol, as it not only provides anxiolysis at low doses but also induces amnesia, has minor hemodynamic effects, and preserves spontaneous breathing. In comparison with midazolam and dexmedetomidine, it is easier to titrate due to its short half-life, and remimazolam is a more profound inducer of amnesia for the procedure than dexmedetomidine. In addition to the plausible advantages of the use of remimazolam in a difficult airway scenario, it should be mentioned that commonly reported adverse effects of remimazolam include headache, dizziness, bradycardia, hypotension, respiratory depression, nausea, and vomiting [13]. Also, delayed emergence, anaphylaxis, and re-sedation after flumazenil reversal were reported in a scoping review on serious adverse events of remimazolam [14].

Regarding topicalization, the DAS technique describes the application of 20-30 sprays of lidocaine 10% (i.e., 200-300 mg lidocaine) spray to the oropharynx, tonsillar pillars, and base of the tongue during inspiration over five minutes. The Trachospray delivers a soft mist spray of local anesthetics in the upper airway. In a comparative in vitro study, it produced an even coverage in the mouth, hypopharynx, and vocal cords with only a small lung fraction [5]. The deposition of local anesthetics in the targeted area for topical anesthesia of the airway was superior for the Trachospray device compared to a lidocaine spray pump, which produced big droplets that deposited mainly at the hypopharynx. In a clinical evaluation, use of the Trachospray device with 2 x 2 ml lidocaine 4% enabled passing of the vocal cords with a flexible bronchoscope without further use of topical local anesthetics in 85% of the volunteers after a small dose of intravenous sufentanil and midazolam [5]. The mean spraying time with the Trachospray device in that study was one minute shorter than the suggested five minutes in the DAS guidelines. Also, the lidocaine dose administered was much lower than in the DAS technique (160 mg vs. 200-300 mg lidocaine) and far lower than the recommended maximal dose of 9 mg/kg (lean body weight) of topical lidocaine.

For the nasal route, the DAS technique recommends the spray of 2.5 ml co-phenylcaine (2.5 ml lidocaine 5%/phenylephrine 0.5%). We used a nasal atomizer to spray 0.5 ml of xylometazoline and 0.5 ml of lidocaine 4% into the right nostril. The DAS guidelines advise testing the topicalization. This was done in our patient with the careful nasal passage of a nasopharyngeal airway lubricated with 2% lidocaine gel.

For the topicalization of the vocal cords, the DAS technique advises 2 ml of 2% lidocaine spray above, at, and below the vocal cords via an epidural catheter or the working channel of a flexible bronchoscope. We administered a similar total dose of lidocaine 1% spray via an epidural catheter. This is in consensus with the DAS guidelines in which it is stated that some studies have shown that lower concentrations of lidocaine are as effective as higher concentrations. The DAS guidelines do not make a statement on using a single-orifice or a multi-orifice epidural catheter. We used a multi-orifice epidural catheter with only sideway orifices. To achieve also a forward spray pattern as with a single-orifice epidural catheter, we cut the last millimeters (the blue tip) of the epidural catheter, resulting in a combined forward-sideway spray pattern.

Although remimazolam is known for its ability to induce amnesia, the minimal dosage needed to prevent recall has not been described. Furthermore, future research should elucidate which dosage is needed for very light sedation or anxiolysis versus deep sedation. Worth mentioning is that in our experience, regardless of the sedation level, patients may lie with their eyes open or seem to be more awake while the comfort level, anxiolysis, and amnesia are good.

Although this case report describes merely one case in which the combination of remimazolam and Trachospray was used during flexible nasal intubation in spontaneously breathing patients, we think that the described techniques may be of value to other patients and healthcare workers. Although "awake" flexible intubation to date is a procedure that is often associated with stress in both patients and anesthesiologists, there are several reasons to choose "awake" tracheal intubation techniques, either via flexible scope or video laryngoscope, with a tube introduced nasally or orally. Traditionally, anatomically difficult airways are considered, but physiologically challenging airways can also be a reason for intubation techniques under excellent topicalization and carefully titrated anxiolysis.

## Conclusions

In this case presentation, the newly developed ultra-short-acting benzodiazepine remimazolam proved suitable for titrated sedation for flexible intubation. It provided adequate anxiolysis, amnesia, and sedation on one hand while ensuring spontaneous breathing on the other. The specifically designed Trachospray device may be of help for topicalization of the upper airway preceding flexible intubation. Still, these findings are based on a single case report, and further research is needed to establish the superiority of any of these techniques in comparison with other accepted methods.
